# Oxidation of 5-methylaminomethyl uridine (mnm^5^U) by Oxone Leads to Aldonitrone Derivatives

**DOI:** 10.3390/biom8040145

**Published:** 2018-11-14

**Authors:** Qishun Zhou, Bao Tram Vu Ngoc, Grazyna Leszczynska, Jean-Luc Stigliani, Geneviève Pratviel

**Affiliations:** 1 CNRS, Laboratoire de Chimie de Coordination, 205 route de Narbonne, 31077 Toulouse CEDEX4, France; qishun.zhou@univ-tlse3.fr (Q.Z.); bao-tram.vu-ngoc@univ-tlse3.fr (B.T.V.N.); jean-luc.stigliani@lcc-toulouse.fr (J.-L.S.); 2 Université de Toulouse, Université Paul Sabatier, UPS, 31330 Toulouse, France; 3 Institute of Organic Chemistry, Faculty of Chemistry, Lodz University of Technology, Zeromskiego 116, 90-924 Lodz, Poland; grazyna.leszczynska@p.lodz.pl

**Keywords:** RNA oxidation, aldonitone, oxaziridine, KHSO_5_, water solvent

## Abstract

Oxidative RNA damage is linked to cell dysfunction and diseases. The present work focuses on the in vitro oxidation of 5-methylaminomethyl uridine (mnm^5^U), which belongs to the numerous post-transcriptional modifications that are found in tRNA. The reaction of oxone with mnm^5^U in water at pH 7.5 leads to two aldonitrone derivatives. They form by two oxidation steps and one dehydration step. Therefore, the potential oxidation products of mnm^5^U in vivo may not be only aldonitrones, but also hydroxylamine and imine derivatives (which may be chemically more reactive). Irradiation of aldonitrone leads to unstable oxaziridine derivatives that are susceptible to isomerization to amide or to hydrolysis to aldehyde derivative.

## 1. Introduction

RNA shows about a hundred different post-transcriptionally modified nucleosides and most of them are in tRNA. They are enzymatically synthesized and are located at specific sites. They play diverse and indispensable roles in the decoding of mRNA and in the gene regulation in all living organisms [1,2]. Modifications in the anticodon stem and loop (ASL) domain of tRNA are the most diverse and the most complex of all modifications in RNA. Because the ASL domain is critical for accurate and efficient translation, the ASL natural modifications directly contribute to the correct decoding of mRNA codons and they constitute an essential key in the regulatory processes of translation [3,4].

Uridine at the wobble anticodon position 34 of tRNAs is often modified with a methylene carbon on C5 of uracil with a variety of modifications such as 5-methylaminomethyl, 5-oxyacetic acid, 5-taurinomethyl. In addition, position 34 uridine may also carry a sulfur or selenium atom on C2 that may be associated to C5 modification and result in hypermodified ribonucleosides, such as 5-methylaminomethyl-2-thiouridine (mnm^5^s^2^U) and 5-methylaminomethyl-2-selenouridine(mnm^5^se^2^U) [4,5,6]. Modifications to position 34 uridines are crucial for the precise decoding of the genetic information and for the tuning of protein synthesis [4,7,8].

The present work deals with the sensitivity to oxidation of the modified ribonucleoside 5-methylaminomethyluridine (mnm^5^U). The 5-methylaminomethyl group on the C5 position of uracil has been found in bacteria and archaea [5,6]. For instance, the modified uridine mnm^5^U is present at the wobble anticodon position 34 in *E. coli* tRNA^Arg4^ isoaceptor [9] and the hypermodified nucleoside, 5-methylaminomethyl-2-thiouridine also at position-34 in *E. coli* tRNA^Lys^ [4,10]. Another related hypermodified nucleoside, 5-methylaminomethyl-2-selenouridine, was found in bacteria [11].

The fact that RNA is sensitive to oxidation is now well documented and oxidative RNA damage has been connected to various forms of cell dysfunction or diseases [12,13,14]. Apart from general RNA oxidation implying normal nucleobases in particular guanines [15,16], recent focus on natural modified RNA bases have provided interesting insights into their high sensitivity to oxidation [17,18,19,20,21]. Hydrogen peroxide, which belongs to the reactive oxygen species of oxidative stress, readily reacts with sulfur-containing nucleobases. Oxidation of 2-thiouridine leads to uridine and/or 4-pyrimidinone derivative, depending on the experimental conditions [17,18,21]. Oxidation of mnm^5^s^2^U leads mainly to mnm^5^U [18]. Therefore, the study of the sensitivity of modified RNA nucleosides to oxidative stress appears relevant for the identification of their endogenous oxidative damage, which might prove biologically significant.

We report oxidative damage of mnm^5^U in the presence of oxone (KHSO_5_), which is an asymmetric peroxide soluble in water at physiological pH. It is convenient for in vitro studies, since its reactivity mimics that of hydrogen peroxide while being more reactive. Besides, its reactivity is similar to that of the biologically relevant asymmetric peroxide, peroxynitrite (ONOO^−^).

The identification of chemical modifications due to oxidation on the 5-methylaminomethyl group at the C5 position of uracil may help better understanding of RNA sensitivity to oxidative stress and its consequences in cells. We characterized the oxidation products of mnm^5^U, namely, the 5-methyl-aldonitrone and the 5-aldonitrone-methyl derivatives, 1 and 2, by UV-visible, mass, and NMR spectroscopy. We also describe the secondary products that may form from these aldonitrones in physiological conditions.

## 2. Materials and Methods

### 2.1. Chemicals

Ribonucleoside was a gift from Prof. Barbara Nawrot, Polish Academy of Sciences, CMMS, Lodz, Poland and Dr. Elzbieta Sochacka, Technical University, Lodz, Poland. It was prepared according to published procedure [22]. Oxone (triple salt K_2_SO_4_, KHSO_4_, 2 KHSO_5_) was purchased from Aldrich, Saint-Louis, MO, USA. The mnm^5^U ribonucleoside was dissolved in ultrapure milliQ water, at a concentration of 10 mM, and it was stored at −20 °C.

### 2.2. Oxidation Reaction

Typical oxidation reactions were carried out in a final volume of 100 µL of 50 mM phosphate buffer (pH 6.5–7.5) with 20 µM mnm^5^U and 40 µM or 2 mM KHSO_5_ and lasted 10 min at room temperature. The addition of 10 µL of 1 M hepes pH 8 buffer stopped the reaction. The reaction was analyzed by High Performance Liquid Chromatography (HPLC).

### 2.3. Chromatography

HPLC analysis was done on a 5 µm C18 reverse phase column (uptisphere 5HDO, 250 × 4.6 mm from Interchim, Montluçon, France) eluted with a gradient of 5 mM ammonium acetate (NH_4_OAc) pH 6.5 aqueous buffer and methanol at a flow rate of 0.5 mL/min. After 5 min at 100% NH_4_OAc buffer, the percentage of methanol increased linearly to 30% in 20 min and jt remained at 30% for 5 min, before returning to the initial conditions. A diode array detector recorded the in-line UV-visible spectra. LC/ESI-MS analyses were carried out in the same liquid chromatography conditions.

### 2.4. Mass Spectrometry

Either direct mass analysis or analysis through coupling with liquid chromatography was performed with the mass spectrometers, low resolution (Q TRAP 2000, Applied Biosystems, Foster City, CA, USA), and high resolution (Q TOF 1er, Waters, Milford, MA, USA) that were operated in the positive and/or negative mode.

### 2.5. Preparation of Aldonitrone Derivatives 1 and 2

Larger scale oxidation of mnm^5^U was performed in a final volume of 300 µL of 50 mM phosphate pH 7.4 buffer, 1 mM mnm^5^U was incubated with 4 mM KHSO_5_. Oxidation lasted 10 min at room temperature. The addition of 30 µL of 1 M hepes pH 8 buffer stopped the reaction. Products 1 and 2 were collected from liquid chromatography on a semi-preparative column and the fractions were lyophilized. The oxidation reaction was repeated three times. The semi-preparative 10 µm C18 reverse phase column (nucleosil, 250 × 10 mm from Interchim, Montluçon, France) was eluted with a gradient of 5 mM ammonium acetate (NH_4_OAc) pH 6.5 aqueous buffer and methanol at a flow rate of 2.4 mL/min. After 5 min at 100% NH_4_OAc buffer, the percentage of methanol increased linearly to 30% in 40 min, maintained at 30% for 5 min, and programmed to go back to initial conditions within 5 min. A diode array detector recorded the in-line UV-vis spectra of the products.

**Aldonitrone 1.**

UV-visible (H_2_O): λ_max_ = 245, 263, and 319 nm.

HR-ESI < 0: [M − H]^−^ *m/z* for C_11_H_14_N_3_O_7_ calcd: 300.0832, obs: 300.0833 amu.

HR-ESI > 0: [M + H]^+^ *m/z* for C_11_H_16_N_3_O_7_ calcd: 302.0988, obs: 302.0994 amu. The nucleobase fragment C_6_H_8_N_2_O_3_ was observed at *m/z* = 170.0586 amu.

^1^H-NMR (600 MHz, D_2_O) (298 K) δ (ppm): 9.79 (s, 1H, H6), 7.78 (s, 1H, H aldonitrone), 5.92 (d, 1H, *J* = 3.8 Hz, H1’), 4.27 (m, 1H, H2’), 4.12 (m, 1H, H3’), 4.07 (m, 1H, H4’), 3,84 (m, 1H, H5’), 3.76 (m, 1H, H5”), 3.75 (s, 3H, methyl).

^13^C-NMR (151 MHz, D_2_O) δ (ppm): 163.2 (C4), 150.6 (C2), 142.2 (C6), 132.5 (aldonitrone), 105.9 (C5), 89.7 (C1’), 73.9 (C2’), 84.3 (C3’), 69.8 (C4’), 61.8 (C5’), 52.4 (CH_3_).

**Aldonitrone 2.**

UV-visible (H_2_O): λ_max_ = 236 and 266 nm

HR-ESI > 0: [M + Na]^+^ *m/z* for C_11_H_15_N_3_O_7_Na calcd: 324.0808, obs: 324.0803 amu. The nucleobase fragment C_6_H_7_N_2_O_3_Na was observed at *m/z* = 192.0381 amu.

^1^H-NMR (600 MHz, D_2_O) (278 K) δ (ppm): 7.91 (s, 1H, H6), 6.70 (d, 1H, ^2^*J* = 6.1 Hz, = CH_2_), 6.52 (d, 1H, ^2^*J* = 6.1 Hz, = CH_2_), 5.61 (m, 1H, H1’), 4.50 (under the signal of water, CH_2_), 4.04 (m, 1H, H2’), 3.93 (m, 1H, H3’), 3.82 (m, 1H, H4’), 3.63 (m, 1H, H5’), 3.52 (m, 1H, H5”).

^13^C-NMR (151 MHz, D_2_O) (278 K) δ (ppm): 164.3 (C4), 151.1 (C2), 142.7 (C6), 134.1 (C = NO), 105.3 (C5), 89.0 (C1’), 74.0 (C2’), 68.7 (C3’), 84.2 (C4’), 60.5 (CH_2_), 60.2 (C5’).

**Hydroxylamine derivative 7.**

UV-visible (H_2_O): λ_max_ = 265 nm.

HR-ESI > 0: [M + Na]^+^ *m/z* for C_10_H_14_N_3_O_7_Na calcd: 312.0808, obs: 312.0805 amu.

^1^H-NMR (600 MHz, D_2_O) (278 K) δ (ppm): 7.66 (s, 1H, H6), 5.61 (m, 1H, H1’), 4.04 (m, 1H, H2’), 3.93 (m, 1H, H3’), 3.82 (m, 1H, H4’), 3,63 (m, 1H, H5’), 3.52 (m, 1H, H5”), 3.44 (m, 2H, CH_2_).

^13^C-NMR (151 MHz, D_2_O) (278 K) δ (ppm): 164.3 (C4), 151.1 (C2), 140.6 (C6), 108.5 (C5), 89.0 (C1’), 74.0 (C2’), 68.7 (C3’), 84.2 (C4’), 49.2 (CH_2_), 60.2 (C5’).

### 2.6. Nuclear Magnetic Resonance

NMR samples were prepared by dissolving the samples in 350 µL of D_2_O using a Shigemi NMR tube from Sigma-Aldrich, Saint-Louis, MO, USA. 1D and 2D ^1^H and ^13^C experiments were recorded on a Bruker Avance I 500 MHz or Bruker NEO 600 MHz spectrometers (Bruker, Billerica, MA, USA). All chemical shifts for ^1^H and ^13^C are relative to TMS using ^1^H (residual) chemical shifts of the solvent as a secondary standard. All the ^1^H and ^13^C signals were assigned on the basis of the chemical shifts, the spin-spin coupling constants, the splitting patterns, and the signal intensities, and by using ^1^H-^13^C HSQC and ^1^H-^13^C HMBC experiments. 1D NOE (Nuclear Overhauser Effect) experiments were realized with a mixing time of 500 ms. Spectra were recorded at 298 K and/or 278 K as indicated.

### 2.7. Light Irradiation of Aldonitrone 1

Irradiation of 300 µL D_2_O solution of 1 was performed with a mercury lamp (Mercury lamp, Oriel, 100 W). The sample was kept in ice during irradiation. Irradiation (1.3 mW/cm²) was performed with a glass filter for 1 h and 30 min. The conversion was almost complete. Aldonitrone 1 transformed to two products in 1:1 ratio. The products of photodecomposition of 1, namely oxaziridine derivatives 3 and 4, were not isolated, but characterized as a 1:1 mixture in solution.

**Oxaziridine** (**3 + 4**) UV-visible (H_2_O): λ_max_ = 269 nm.

**Oxaziridine** (**3**) HR-ESI > 0: [M + H]^+^ *m/z* for C_11_H_16_N_3_O_7_ calcd: 302.0988, obs: 302.1002 amu. The nucleobase fragment C_6_H_8_N_2_O_3_ was observed at *m/z* = 170.0575 amu. [M + Na]^+^ *m/z* for C_11_H_15_N_3_O_7_Na calcd: 324.0808, obs: 324.0800.

**Oxaziridine** (**4**) HR-ESI > 0: [M + H]^+^ *m/z* for C_11_H_16_N_3_O_7_ calcd: 302.0988, obs: 302.1003 amu. The nucleobase fragment C_6_H_8_N_2_O_3_ was observed at *m/z* = 170.0577 amu. [M + Na]^+^ *m/z* for C_11_H_15_N_3_O_7_Na calcd: 324.0808, obs: 324.0805.

**Oxaziridine** (**3 + 4**) ^1^H-NMR (600 MHz, D_2_O) (278 K) δ (ppm): 7.76 (s, 1H, H6, 3 or 4), 7.74 (s, 1H, H6, 3 or 4), 5.59 (m, 2H, H1’, 3 and 4), 4.50 (under signal of water, CH oxaziridine 3 and 4), 3.98 (m, 2H, H2’, 3 and 4), 3.90 (m, 2H, H3’, 3 and 4), 3.80 (m, 2H, H4’, 3 and 4), 3.65 (m, 2H, H5’, 3 and 4), 3.51 (m, 2H, H5”, 3 and 4), 2.56 (s, 6H, methyl, 3 and 4).

**Oxaziridine** (**3 + 4**) ^13^C-NMR (151 MHz, D_2_O) (278 K) δ (ppm) from HSQC and HMBC experiment: 164.5 (C4), 150.8 (C2), 140.6 (C6), 107.9 (C5), 89.3 (C1’), 75.7 (CH oxaziridine), 73.9 (C2’), 68.4 (C3’), 83.6 (C4’), 57.9 (C5’), 46.9 (CH_3_).

### 2.8. Modeling

The geometry of the structures was optimized at the M06-2X/6-311++G(d,p) model chemistry with the GAUSSIAN 09 program [23]. The bulk solvent effects were described with the integral equation formalism polarizable continuum Model (IEFPCM) with water as solvent [24]. Vibrational frequencies were computed to confirm the convergence to local minima and to calculate the unscaled zero-point-energy (ZPE) and the Gibb’s free energy at 298 K.

## 3. Results

### 3.1. Oxidation of 5-methylaminomethyl uridine Leads to Two Oxidation Products

**Figure 1:**
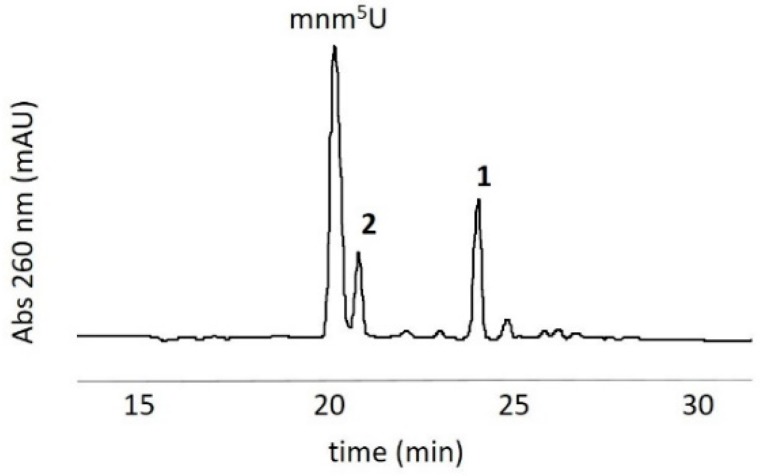
HPLC trace of the oxidation of 5-methylaminomethyl uridine (mnm^5^U) (20 µM) with KHSO_5_ (2 mM) for 10 min in 50 mM phosphate buffer at pH 7.5. Conversion 50%.

**Figure 2:**
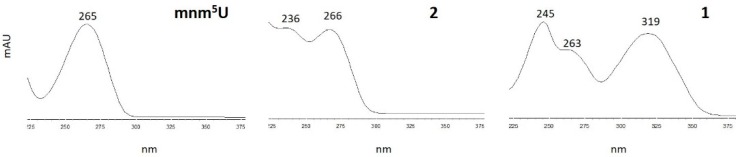
In-line UV-visible spectra (220–400 nm) of Figure 2: 5-methylaminomethyl uridine (mnm^5^U) (Rt = 19 min), 2 (Rt = 21 min), and 1 (Rt = 24 min).

Incubation of 20 µM mnm^5^U, in pH 7.5 phosphate buffer, for 10 min at room temperature, with KHSO_5_ (2 mM) afforded two oxidation products, 1 and 2. The HPLC analysis of the reaction is shown in Figure 1 and Figure 2. The starting ribonucleoside eluted at a retention time of 19 min, and 1 and 2 at 24 and 21 min, respectively. According to the decrease of the HPLC area of the peak of initial mnm^5^U, the reaction resulted in ~50% conversion of mnm^5^U.

To identify the oxidation products, the reaction was performed at higher concentration of mnm^5^U (1 mM) and KHSO_5_ (4 mM) in 50 mM phosphate pH 7.5 buffer for 10 min. Products of the reaction were collected separately and lyophilized.

### 3.2. Characterization of Oxidation Product of mnm^5^U: Aldonitrone 1

**Scheme 1:**
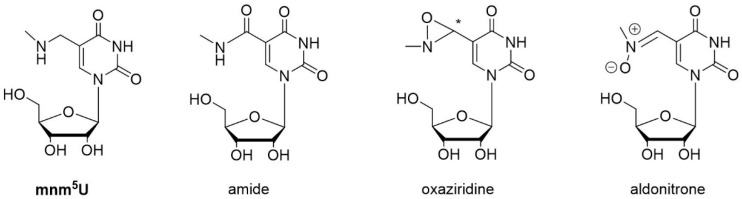
Possible oxidation products of 5-methylaminomethyl uridine (mnm^5^U) with a mass increase of 14 amu with respect to mnm^5^U.

Product 1 proved to be stable during isolation. High-resolution negative electrospray mass analysis showed a [M − H]^−^ ion with *m/z* = 300.0833 corresponding to C_11_H_14_N_3_O_7_, *m/z* (calcd) = 300.0832 amu (Figure S1). The molecular mass of 1 is 301 g/mol, which corresponds to an increase of 14 amu with respect to the molecular mass of the initial mnm^5^U ribonucleoside. With such a molecular mass, compound 1 might be either an aldonitrone, an oxaziridines or an amide derivative that are three isomers with the same molecular formula, and be susceptible to isomerization through the oxaziridine derivative (Scheme 1) [25,26]. Indeed, the oxidation of secondary amine by oxygen atom donors (hydroperoxides, dioxirane, peracids, oxone) may lead to aldonitrone, oxaziridine, and/or amide derivative. Nitrones are mainly prepared by oxidation of hydroxylamines [27,28]. They may also be obtained from oxidation of imines [29] and amines [30,31,32,33]. Usual oxidants are oxygen atom donors, such as hydroperoxides (including H_2_O_2_) activated by metal ions, peracids, and dioxirane. However, oxaziridines are the most common oxidation products of imines with a variety of reagents [26,34,35,36,37]. On the other hand, oxidation of primary amines by oxone has been reported to afford nitrones [38,39,40]. Up to now, literature reports deal with oxidations that are not performed in water.

**Figure 3:**
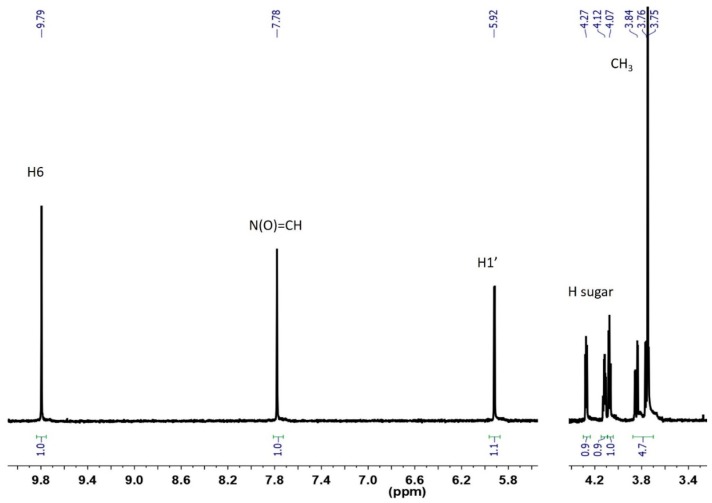
^1^H-NMR (600 MHz) spectrum of 1 in D_2_O at 298 K.

The observed strong π−π* band at 319 nm on UV-visible spectrum of 1 (Figure 2) is compatible with an *N*-alkyl aldonitrone function as shown in Scheme 1 [34,41,42]. The aldonitrone structure of 1 was unambiguously determined by NMR. The 600 MHz ^1^H-NMR spectrum of 1 in D_2_O is shown in Figure 3. To make it simple, the numbering of atoms corresponds to that of uridine. Oxidation product 1 is a ribonucleoside with the sugar protons showing typical resonances at δ 5.92 ppm for H1’ (^3^*J*_HH_ = 3.5 Hz) and between 4.27 and 3.76 ppm for H2’, H3’, H4’, H5’, and H5”. One methyl group can be observed at δ 3.75 ppm (accounting for 3 H). Two downfield singlet resonances at δ 7.78 ppm and δ 9.79 ppm, each of them integrating for 1 H, were attributed to the H of an aldonitrone function and to the H6 proton of nucleobase, respectively, from HSQC and HMBC experiments, as detailed below. From HSQC the protons δ 7.78 ppm (H aldonitrone) and δ 9.79 ppm (H6) correlated through ^1^*J*_CH_ with the carbons δ 132.5 ppm (C aldonitrone) and δ 142.2 ppm (C6), respectively. From HMBC data the protons of the methyl group (δ 3.75 ppm) correlated with the carbon δ 132.5 ppm through ^3^*J*_CH_. Therefore, we attributed the H signal δ 7.78 ppm, connected to the carbon signal δ 132.5 ppm (^1^*J*_CH_), to the H atom of the aldonitrone function. Furthermore, in HMBC experiment both H signals at δ 7.78 ppm and at δ 9.79 ppm showed a ^3^*J*_CH_ correlation with the carbon δ 163.2 ppm (attributed to the C4 of mnm^5^U by analogy with reported NMR of thymine as described in the literature) while only the δ 9.79 ppm correlated with the carbon δ 150.6 ppm (attributed to the C2 of mnm^5^U) (Figure S2A). The proton at δ 9.79 ppm also showed a ^3^*J*_CH_ correlation with the carbon δ 132.5 ppm, which is in accordance with the fact that it corresponds to the H6 proton of modified uracil.

**Scheme 2:**
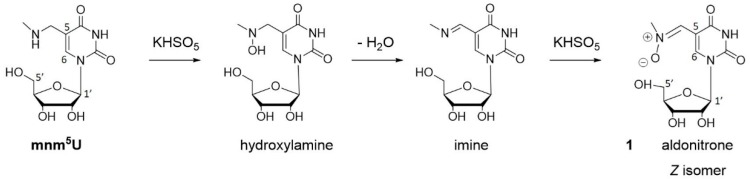
Proposed mechanism of oxidation of 5-methylaminomethyl uridine (mnm^5^U) with oxone: formation of the stable product 1. Numbering of atoms corresponds to the numbering of uridine.

The mechanism of formation of product 1 is proposed in Scheme 2. The secondary amine of mnm^5^U is oxidized by KHSO_5_ to the intermediate hydroxylamine derivative. The loss of a molecule of water leads to the imine, which is oxidized by a second molecule of KHSO_5_.

Only one methyl signal and only one azomethine proton was observed in the ^1^H-NMR spectrum. This probably implies that only one aldonitrone stereo isomer formed in the reaction. The NOE correlation between the methyl group (δ 3.75 ppm) and the H proton of aldonitrone (δ 7.78 ppm) unambiguously assigns the *Z* configuration, which is the preferred configuration of aldonitrones (Figure S2B). The deshielding effect of aldonitrone oxygen in the *Z*-geometry causes a lower field absorption of the nucleobase H6 proton of 1, δ 9.79 ppm, with respect to the H6 of mnm^5^U δ 8.35 ppm [9].

**Figure 4:**
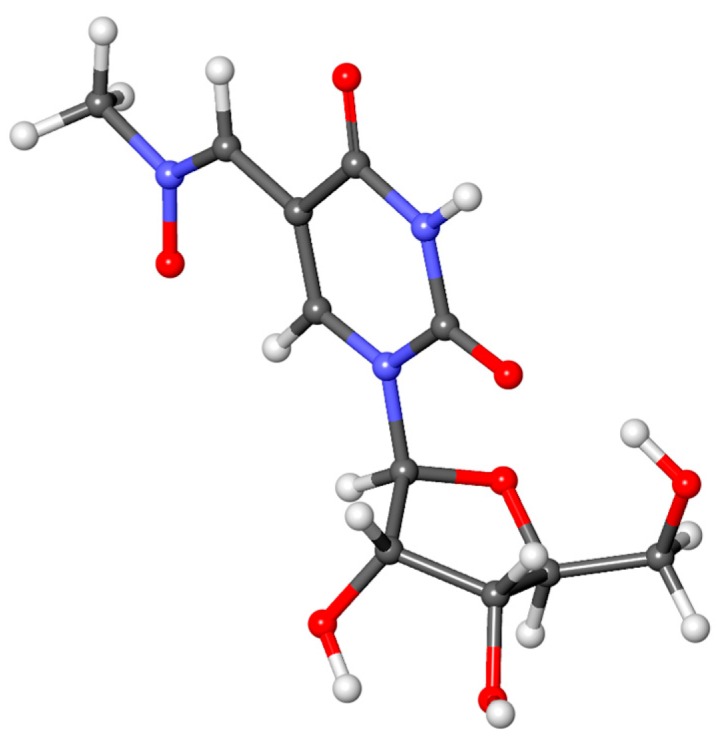
Optimized geometry of (*Z*) aldonitrone 1 showing a planar arrangement for optimal electron delocalization between aldonitrone moiety and nucleobase.

Molecular modeling confirms the lower energy of the Z as compared to the E isomer. The Z conformation shows a planar geometry optimizing electron delocalization between the aldonitrone function and the aromatic ring of nucleobase (Figure 4). The E conformation is less favorable due to steric hindrance between the methyl group and the H6 proton of the nucleobase.

The proposed structure of *N*-methyl-aldonitrone 1 is compatible with previously published NMR data for aryl-conjugated aldonitrones [38,43,44].

Oxaziridines (Scheme 1) are often reported in the literature as the products of imine oxidation by peracids [34,35,37]. During the oxidation of mnm^5^U with oxone, *N*-methyl-oxaziridine derivative did not form as no NMR signal was observed at the typical chemical shift of the CH proton of oxaziridines, namely around δ 4–5 ppm [45]. Previous use of oxone for secondary amine oxidation also led to nitrone [33,38,39,40].

### 3.3. Photochemical Characterization of Aldonitrone 1

**Figure 5:**
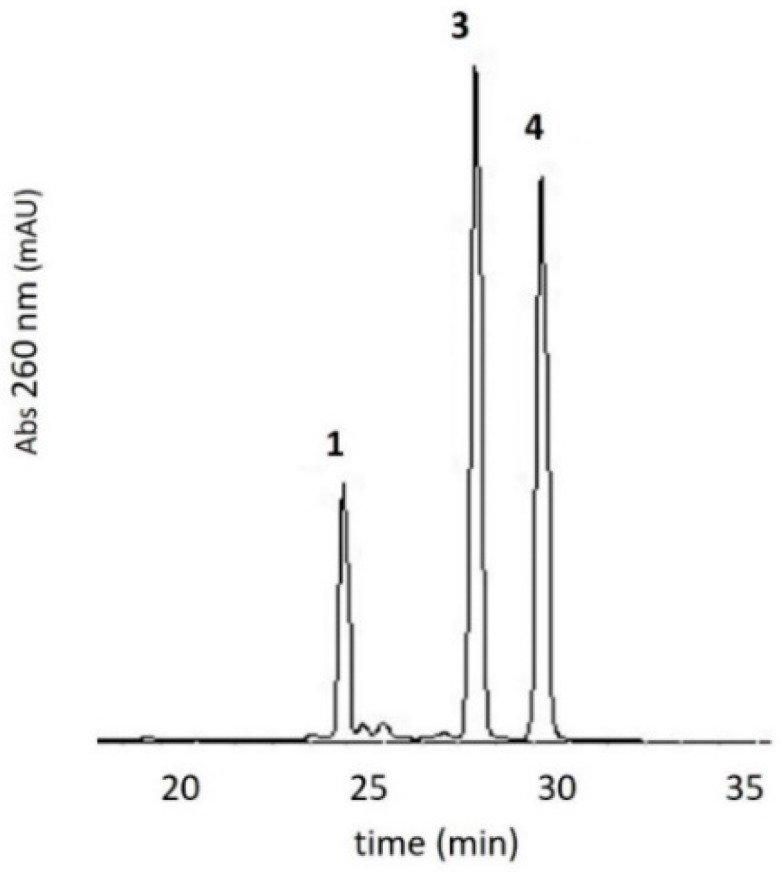
HPLC trace (detection 260 nm) of irradiation of a solution of isolated 1 in water during 30 min showing the two products 3 and 4 at Rt = 27 min and 29 min, respectively.

**Figure 6:**
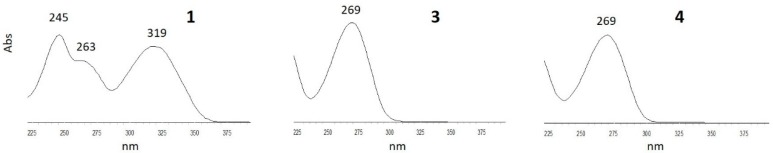
In-line UV-visible spectra (220–400 nm) corresponding to Figure 5: 1 (Rt = 24 min), 3 (Rt = 27 min), and 4 (Rt = 29 min).

Nitrones are known to transform to oxaziridines when irradiated [25,37,41,46,47,48]. Therefore, isolated aldonitrone 1 was exposed to light in H_2_O in an ice bath, with a glass filter (λ cut-off 300 nm). The reaction was followed by HPLC. Aldonitrone 1 transformed to two products 3 and 4 (in about equimolar amount) that eluted at Rt = 27 and 29 min, respectively. The reaction was almost complete within 30 min (Figure 5 and Figure 6). Exposure of 1 to day light on the laboratory bench led to the same products (50% of conversion of 1 after 1h at room temperature) (not shown).

The in-line high resolution mass analysis of the two compounds (3 and 4) reveals that both have the same molecular mass as 1, 301 g/mol, and the same molecular formula, C_11_H_15_N_3_O_7_ (Figure S3).

**Figure 7:**
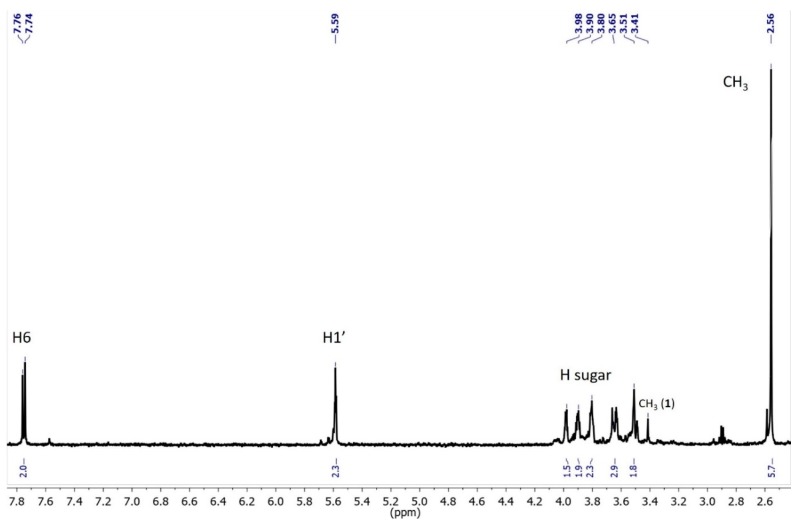
^1^H-NMR (600 MHz, 278 K) spectrum of irradiated 1 in D_2_O. The tube contains 90% of oxaziridines 3 and 4 in a 50:50 ratio and 10% of 1. The solvent peak at δ 4.7 ppm is suppressed.

Irradiation of an NMR tube containing 1 in D_2_O during 1 h and 30 min in ice allowed us to characterize the mixture of the two products (3 and 4) by NMR. The ^1^H spectrum of 3 + 4 mixture (1:1 ratio) in D_2_O at 278 K is shown in Figure 7. The conversion of 1 into 3 + 4 was almost complete, since the signals of aldonitrone accounted for only 10% (as deduced from comparison of the methyl of the starting product 1 δ 3.41 ppm with the methyl of the new products δ 2.56 ppm).

From the NMR analyses 3 and 4 were identified as two oxaziridine derivatives. Two singlet signals at δ 7.76 ppm and δ 7.74 ppm were assigned to two H6 protons of nucleobases (each integrating for 1H). The H1’ proton appeared as a broad signal at δ 5.59 ppm (integration 2H). Ribose protons (10 H) were found between δ 3.91 ppm and δ 3.51 ppm. Finally, a singlet accounting for 6 H was observed at δ 2.56 ppm and it was attributed to the methyl groups. Thus, although 3 and 4 showed two distinct resonances for H6 and H1’, two separate methyl groups could not be identified, only one singlet accounting for 6 H appeared at δ 2.56 ppm.

NOE experiment on the 3 + 4 mixture showed a correlation between the H6 protons (δ 7.76 and 7.74 ppm) and some protons, the resonances of which appeared under the signal of water (two singlets at δ ~ 4.5 ppm). Another NOE correlation was also noted between the protons of the methyl group (δ 2.56 ppm) and the same protons under the signal of water. These two hidden ^1^H resonances that were located under the water signal were attributed to the CH proton of the oxaziridine function. The chemical shift of the CH proton of oxaziridines had been previously reported as being around δ 4–5 ppm [45], which is in accordance with the present data for 3 and 4.

**Scheme 3:**
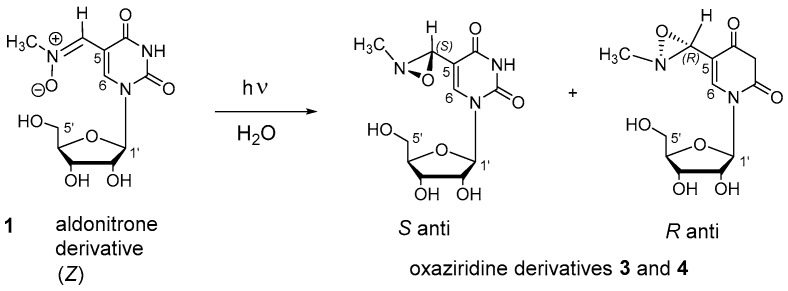
Formation of two epimers of oxaziridine, derivatives 3 and 4, in about equimolar amount, upon irradiation of aldonitrone 1. Numbering of atoms corresponds to the numbering of uridine.

Furthermore, as deduced from the correlation between the methyl and the hidden H-atom, the H atom of oxaziridine, and the methyl group are on the same side of the oxaziridine cycle. This implies that among the four possible isomers, the two observed oxaziridines are in *anti* conformation (Scheme 3).

A 2D ^1^H/^13^C HSQC NMR experiment assigned the C6 carbons of the two nucleobases (δ 140.6 ppm) connected to H6 protons (δ 7.76 and 7.74 ppm), the C1’ carbons (δ 89.4 ppm) connected to H1’ protons (δ 5.6 ppm), the methyl carbons (δ 46.9 ppm) connected to methyl protons (δ 2.56 ppm), and new carbons (δ 75.7 ppm) connected to hidden protons (δ ~ 4.5 ppm).

Furthermore, weak ^1^H/^13^C HMBC correlations could be detected between the protons δ ~ 4.5 ppm and the C6 carbons δ 140.6 ppm, the methyl carbons δ 46.9 ppm, and carbons δ 108.0 ppm (C5 of nucleobase). Similarly, the carbons δ 75.7 ppm showed HMBC correlations with the H6 and methyl protons. These data confirm the proposed oxaziridine function on the uridine substituent. The ^13^C and ^1^H resonances of the sugar moiety were also unambiguously assigned.

**Figure 8:**
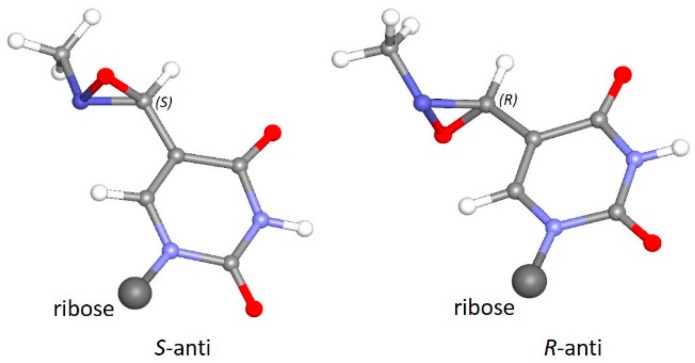
Geometry optimization of oxaziridine derivatives 3 and 4.

Thus, irradiation of aldonitrone 1 led to the mixture of two *anti* diastereoisomers of oxaziridines, S-anti and R-anti (Scheme 3 and Figure 8). S and R refer to the configuration of the carbon atom of oxaziridine. The configuration of the nitrogen atom of oxaziridine is blocked. The reaction was almost complete (yield 90%) after 1 h and 30 min in ice.

### 3.4. Stability of Oxaziridines 3 and 4 in the Dark

**Figure 9:**
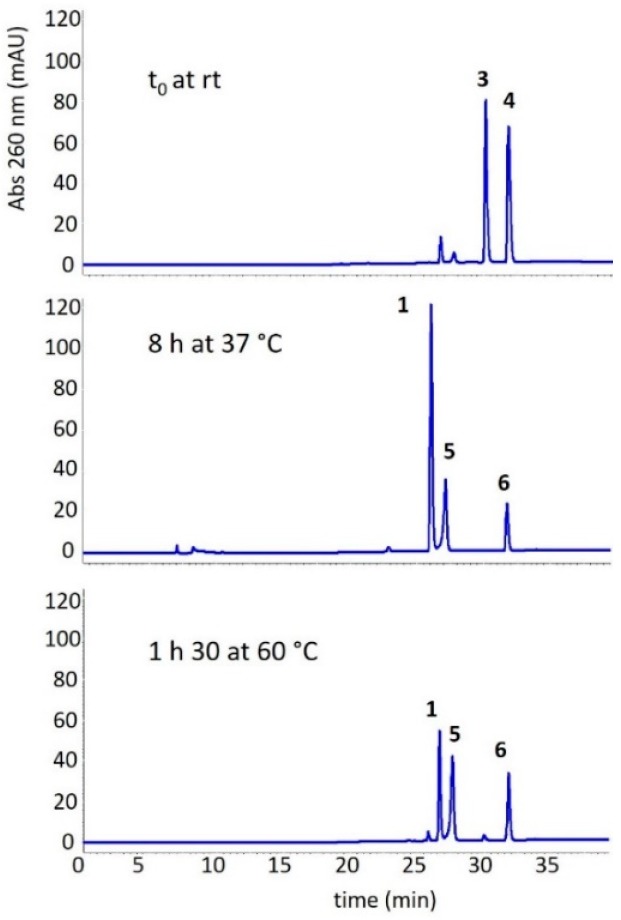
HPLC analysis of the transformation of oxaziridines 3 and 4 back to 1 in the dark. Upper trace, at t0 the mixture of 3 and 4 at Rt = 30 min and 32 min, respectively. Middle trace, 1 (Rt = 26 min) is the major product. It is associated with the aldehyde derivative (5) (Rt = 27 min) and the amide derivative (6) (Rt = 32 min). Lower trace, the proportion of 5 and 6 becomes more important with respect to 1 (Rt = 26 min). rt: room temperature.

**Figure 10:**
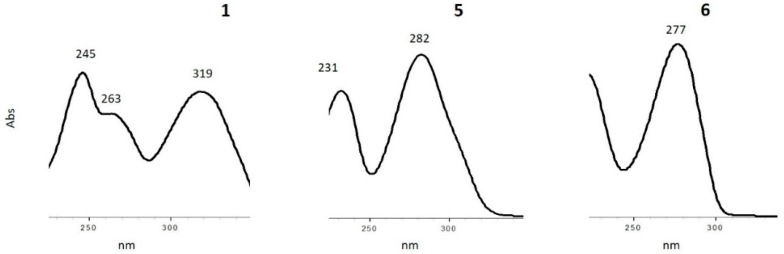
In-line UV spectra (220–350 nm) corresponding to Figure 9, compounds 1, 5, and 6.

After incubation of the NMR tube containing oxaziridines 3 + 4 mixture in water in the dark in the fridge for three weeks the NMR spectrum corresponded to the one of pure initial aldonitrone 1. Therefore, photo-isomerization of 1 is completely reversible in the dark at ~5 °C. Thermal isomerization of oxaziridines to nitrone and/or amide is a common property of these cyclic compounds [25,49]. Incubation at a higher temperature (37–60 °C) of the 3 + 4 mixture gave rise not only to 1, but also to aldehyde (5) and amide (6) derivatives. The percentage of aldehyde and amide increased with temperature (Figure 9 and Figure 10). The products were identified by LC-ESI-MS analysis.

**Scheme 4:**
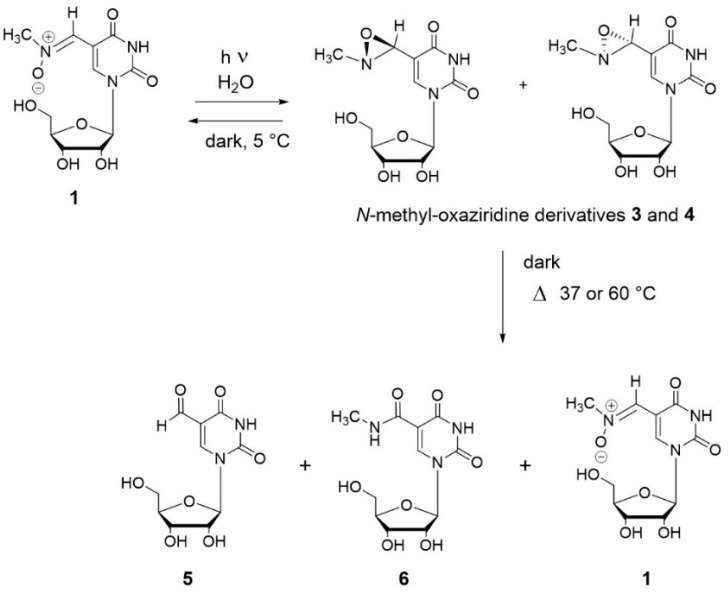
Stability of oxaziridines 3 and 4 in the dark. At low temperature only isomerization to aldonitrone is observed. Upon heating isomerization to amide 6 and hydrolysis of oxaziridines to the aldehyde derivative 5 is observed together with back isomerization to aldonitrone 1.

The mixture of oxaziridines 3 and 4 was incubated in water in the dark at 37 or 60 °C. Compounds 3 and 4 transformed (principally) to aldonitrone 1 and to two other products (5 and 6). Aldonitrone 1 in-line mass spectrum (positive electrospray) showed signals at *m/z* = 324.0808 amu, and *m/z* = 170.0565 amu, which correspond to the sodium adduct of general formula C_11_H_15_N_3_O_7_Na (1) and to the nucleobase fragment C_6_H_8_N_3_O_3_, respectively. The in-line mass spectrum of 5, the aldehyde derivative, showed signals at *m/z* = 295.0544 amu, and *m/z* = 141.0299 amu, which correspond to the sodium adduct of general formula C_10_H_12_N_2_O_7_Na and to the nucleobase fragment C_5_H_5_N_2_O_3_, respectively. Finally, the in-line mass spectrum of 6, the amide derivative, showed signals at *m/z* = 324.0807 amu, and *m/z* = 170.0567 amu, that correspond to the sodium adduct of general formula C_11_H_15_N_3_O_7_Na and to the nucleobase fragment C_6_H_8_N_3_O_3_, respectively (Figure S4). Both aldehyde and amide are common products of hydrolysis (aldehyde 5) or isomerization (amide 6) of aldonitrone (Scheme 4).

The kinetic of the reaction was faster at 60 °C (total transformation of 3 + 4 in 1 h) as compared to 37 °C (total reaction after 15 h) (Figure 10). The ratio between the three products varied depending on the temperature with the formation of 5 and 6 being favored at higher temperature in accordance with literature [25]. On the other hand, aldonitrone 1 proved stable upon incubation in water at 90 °C in the dark for 5h and stable for 1h in acidic conditions (0.1 M acetic acid in water) at room temperature in the dark. Therefore, while aldonitrone 1 is a stable compound in the dark, oxaziridines 3 and 4 undergo isomerization (to 1 at low temperature and to amide 6 at higher temperature) and/or hydrolysis (aldehyde 5).

### 3.5. Characterization of Oxidation Product of mnm^5^U: Aldonitrone 2

**Figure 11:**
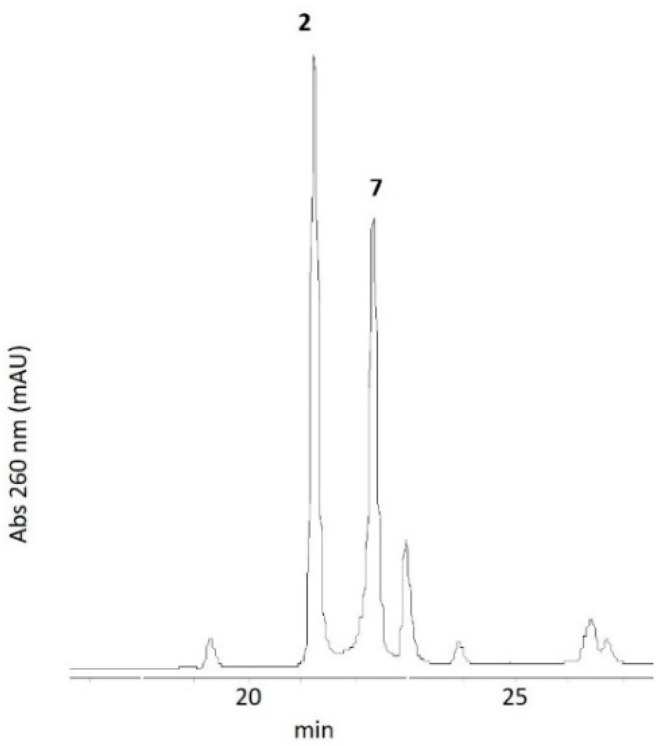
HPLC trace of isolated 2 (Rt = 21 min) showing one major degradation product (7) and a minor one, at Rt 22 and 22.5 min, respectively.

**Figure 12:**
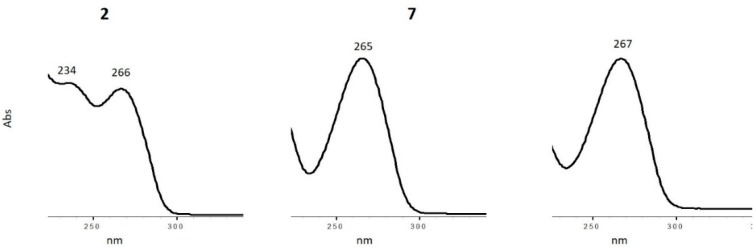
In-line UV-visible spectra (220–350 nm) of chromatogram of Figure 11. From left to right: 2 (Rt = 21 min), degradation product 7 at Rt = 22 min and a minor product at Rt = 22.5 min.

The second oxidation product of mnm^5^U ribonucleoside, product 2 (Figure 1), was isolated by LC chromatography. Unfortunately, it was not stable during the lyophilization process. It partially transformed into a major product (Rt = 22 min) (referred to as 7) (Figure 11 and Figure 12). A minor product was also observed at Rt = 22.5 min.

LC-ESI/MS analysis (high resolution) allowed for us to identify product 2 as an isomer of aldonitrone 1 with a molecular mass of 301 g/mol and the same molecular formula C_11_H_15_N_3_O_7_. Indeed, positive ionization spectrum showed the sodium adduct of the molecular ion [M + Na]^+^ ion C_11_H_15_N_3_O_7_Na calcd: 324.0808, obs: *m/z* = 324.0803 amu (Figure S5). Besides, the molecular mass of 7 was 289 g/mol. It corresponds to a loss of 12 amu as compared to the molecular mass of 2 (Figure S5). The observed signal at *m/z* = 312.0805 amu corresponds to the molecular formula C_10_H_15_N_3_O_7_Na calcd: 312.0808 and thus to the sodium adduct of the molecular ion [M + Na]^+^.

**Scheme 5:**
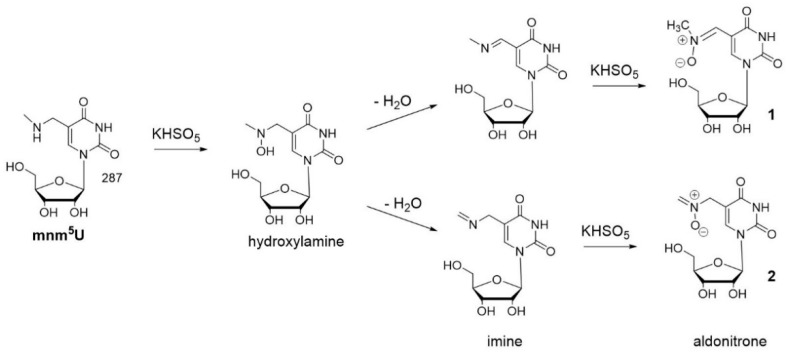
Mechanism of oxidation of mnm^5^U. Formation of two different aldonitrone derivatives (1 and 2).

Compound 2 is likely to be the terminal aldonitrone (Scheme 5) and may form by a similar mechanism with 2 mol. equiv. of KHSO_5_ with respect to mnm^5^U. The loss of a molecule of water from the common intermediate hydroxylamine may occur on either side, which will lead to two isomeric imines. A second oxidation step gives rise to either the *N*-methyl-aldonitrone 1 or the terminal aldonitrone 2.

**Scheme 6:**
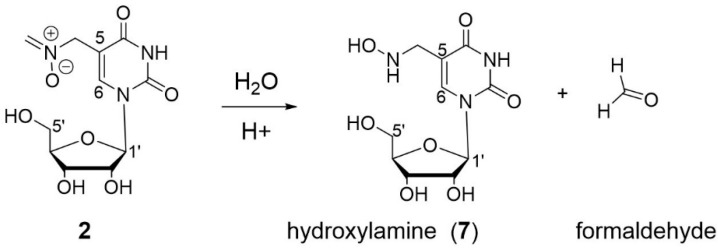
Instability of terminal aldonitrone 2 toward hydrolysis (in acidic conditions). Formation of hydroxylamine derivative, referred to as 7 and formaldehyde. Numbering of atoms corresponds to the numbering of uridine.

Before any more precise characterization (i.e., NMR), arguments in favor of 2 being the terminal aldonitrone are: (i) the UV-vis spectrum of 2 shows a strong absorption band at 236 nm (Figure 2 and Figure 12). While conjugated aldonitrones (such as 1) show a typical absorption band in the 300 nm region,non-conjugated aliphatic aldonitrones show a typical band for the aldonitrone function at λ_max_ c.a. 236 nm [50]; (ii) 2 is, not surprisingly, less stable than 1. Its major degradation product 7, with a molecular mass of 289 g/mol may correspond to the hydroxylamine derivative (Scheme 6); and, (iii) terminal aldonitrones have been previously observed in the literature [28,43].

**Figure 13:**
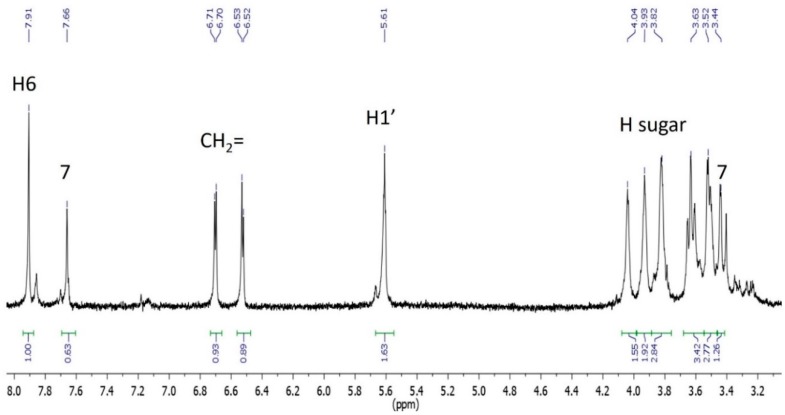
^1^H-NMR (600 MHz, 278 K) spectrum of isolated 2 in D_2_O. The tube contains a mixture of 2 (60%) and 7 (40%). The solvent peak at δ 4.7 ppm is absent.

^1^H-NMR spectrum of isolated 2 (which consists in a mixture of 2 and 7) clearly shows two doublets δ 6.70 and 6.52 ppm with a coupling constant of 6.1 Hz for the two non-equivalent protons of the CH_2_ of the terminal aldonitrone (Figure 13). The methylene CH_2_, which is directly connected to the C5 of the nucleobase, was evidenced by the HSQC experiment showing that a proton signal hidden under the water signal correlated with a carbon δ 60.5 ppm through ^1^*J*_CH_ (Figure S6A). Furthermore, in HMBC experiment this proton signal correlated with carbons C4 δ 164.3 ppm, C6 δ 142.7 ppm, and C5 δ 105.3 ppm of nucleobase (Figure S6B). Product 2 is a ribonucleoside with the H6 nucleobase proton resonance at δ 7.91 ppm, the H1’ at δ 5.61 ppm, and other Hs of sugar between 4.04 and 3.52 ppm. Besides, the ^1^H-NMR spectrum exhibits two additional resonances that belong to product 7, the singlet δ 7.66 ppm, and the multiplet δ 3.44 ppm, attributed to the H6 of the nucleobase and the methylene CH_2_ for ribonucleoside 7, respectively. The ratio between 2 and 7 (measured by integration of the H6 resonances) corresponds to the one observed by UV detection in Figure 11, about 60% and 40%, respectively. Other protons of ribonucleoside 7 show the same chemical shifts as those of 2 (Figure 13: the H1’ δ 5.61 ppm accounts for 1.6 H).

As deduced from the HMBC experiment, the carbon atoms of ribonucleosides 2 and 7 are similar except the C5, which carries the 5-substituent, and was observed at δ 105.3 ppm and 108.5 ppm, respectively. A small difference was also noted between δ 142.7 ppm for the C6 of 2 and δ 140.6 ppm for the C6 of 7 (Figure S6B).

Additionally, a NOE correlation exists between one of the protons of the terminal methylene of 2 δ 6.70 ppm and the signal under the solvent peak (δ 4.5 ppm) attributed to the other CH_2_ group (Figure S6C). Not surprisingly, the configuration of aldonitrone is blocked.

A NOE correlation observed between the CH_2_ protons at C5 of 7 δ 3.44 ppm and the H6 of 7 δ 7.66 ppm also corroborated the attribution of these two NMR resonances to product 7 (Figure S6C).

Overall, the NMR data are in accordance with the proposed structures for terminal aldonitrone 2 and its hydroxylamine hydrolysis product 7.

The stability of 2 in the dark was tested in water under neutral conditions. Ribonucleoside 2 was incubated at 50 °C in water for 4 h. It proved stable (not shown). Therefore, the observed hydrolysis (to hydroxylamine 7) during the lyophilization process was attributed to some transient acidic conditions due to the concentration of the HPLC buffer (ammonium acetate) in the collected fractions [51].

## 4. Discussion

The secondary amine function of the C5 substituent in 5-methylaminomethyl uridine is sensitive to oxidation. Granted that 5-methylaminomethyl modified ribonucleosides are present at the key position 34 in ASL in tRNAs, the present work investigated the oxidation of mnm^5^U, since oxidative degradation of mnm^5^U might have biological relevance and subsequently may disturb biological processes involving tRNAs. Indeed, any chemical modification of the 5-methylaminomethyl group may alter the recognition and binding processes during the translation. Any chemically reactive function appearing during oxidation may lead to further chemical transformation of this substituent or to cross-linking reaction with biological partners.

Oxidation of the mnm^5^U ribonucleoside in water and at pH 7.5 by oxone gave rise to two oxidation products, the *N*-methyl-aldonitrone 1 and the terminal aldonitrone 2 (Figure 1 and Scheme 5). The conversion was 50% after a 10 min reaction at room temperature. According to absorbance at 260 nm, which should be similar for both compounds, the ratio *N*-methyl aldonitrone/terminal aldonitrone is about 2/1 (Figure 1). The *N*-methyl-aldonitrone 1 is thermodynamically preferred to the terminal aldonitrone 2.

Both compounds arise from two successive oxidation reactions and one dehydration process at the secondary amine function of the C5 substituent of uracil base (Scheme 5). The proposed intermediate products, hydroxylamine and imine, were not observed under the experimental conditions used (excess of KHSO_5_ or 4 mol. equiv. KHSO_5_). Thus, after the first oxidation step, the reaction goes quickly.

Both aldonitrones, 1 and 2, proved stable at various tested temperatures in pH 7.5 aqueous buffer. However, in biological medium, nitrones might react with nucleophiles and/or coordinate transition metal ions leading to cross-links of modified tRNA with other biomolecules [40]. Furthermore, nitrones have drawn special attention due to their spin-trapping properties [52,53]. They can react with transient free radical to form more persistent radical paramagnetic species.

The intermediate derivatives (Scheme 5), hydroxylamine and imine, are potentially reactive species in physiological conditions. Hydroxylamines are strong nucleophiles while imines are electrophiles. Various alkylhydroxylamines have been reported to be mutagenic in several bacterial systems [54].

In addition to the direct oxidation products of mnm^5^U mentioned above, other possible oxidation-derived products might form in vivo, such as the oxaziridine derivatives (for instance 3 and 4) (Scheme 3) observed upon light irradiation of aldonitrone 1 (Figure 5). Irradiation of 2 was not studied but should also lead to the corresponding oxaziridines since the light-dependent isomerization of aldonitrones is rather classic [37,41,46,47,48]. As cyclic constrained compounds oxaziridines are less stable than aldonitrones. The half-life of 3 and 4 in water in the dark and at 37 °C was about 3 h (not shown). They spontaneously reverse to aldonitrone, or transform to amide (isomerization) and/or aldehyde (hydrolysis) derivatives (Scheme 4). The aldehyde derivative (5) may be a chemically reactive lesion in vivo. The known reactivity of oxaziridine function in oxygen atom transfer and nitrogen atom transfer reactions should not apply to the present derivatives, since they are not “activated” with electron withdrawing groups [35,37,55,56]. However, we did not test the reactivity of 3 and 4 with amines or sulfides.

Therefore, the present work points out that the oxidation of 5-methylaminomethyl uridine (mnm^5^U) may be possible in vivo under oxidative stress conditions with subsequent potential cellular dysfunction related to altered tRNAs. The main products of oxidation are two aldonitrone derivatives (1 and 2). If the oxidation to aldonitrone is not complete, hydroxylamine and imine intermediate products (Scheme 5) may react with biological molecules. Furthermore, another type of lesion may arise from aldonitrones, like oxaziridines (3 and 4 from 1) (light promoted isomerization), which in turn may transform to amide or aldehyde products. All of these RNA lesions will probably alter tRNA binding and most of them may prove chemically reactive.

## 5. Conclusions

The mnm^5^U ribonucleoside is oxidized by oxone to two oxidation products (1 and 2), the conversion was 50% in pH 7.5 aqueous buffer and room temperature within 10 min. The *N*-methyl-aldonitrone 1 and the terminal aldonitrone 2 are two aldonitrones isomers. Both arise from two successive oxidation reactions (and one dehydration process) at the secondary amine function of the C5 substituent of the uracil base. Further evidence for the structure of 1 was obtained by light irradiation experiments showing its conversion to oxaziridine isomers 3 and 4.

Both aldonitrones 1 and 2 are relatively stable, but oxaziridines 3 and 4 are not. Oxidation of the initial secondary amine function of mnm^5^U is thus possible in vivo and it may lead to lesions, such as aldonitrone, hydroxylamine, imine, oxaziridine, amide, and aldehyde derivatives. None of them looks neutral regarding tRNA in biology.

## Supplementary Materials

The following are available online at http://www.mdpi.com/2218-273X/8/4/145/s1, Figure S1: HR-ESI-MS analysis of 1, Figure S2: NMR analysis of 1: (A) HMBC, (B) NOESY, Figure S3: HR-ESI-MS analysis of 3 and 4, Figure S4: HR-MS analysis of 5 and 6, Figure S5: HR-MS analysis of 2 and its hydrolysis product 7, Figure S6: NMR analysis of 2 + 7: (A) HSQC, (B) HMBC, (C) NOESY.
