# Supplementary material for: Oxidation of 5-methylaminomethyl uridine (mnm5U) by Oxone Leads to Aldonitrone Derivatives
Source: Biomolecules. 2018 Nov 14;8(4):145. doi: 10.3390/biom8040145 (PMC6315764; doi:10.3390/biom8040145)
Supplement: Supplementary file 1 [file biomolecules-08-00145-s001.pdf]

SUPPORTING INFORMATION

Oxidation of 5-methylaminomethyl uridine (mnm⁵U) by oxone leads to aldonitrone derivatives

Qishun Zhou, Bao Tram Vu Ngoc, Grazyna Leszczynska, Jean-Luc Stigliani , and Geneviève Pratviel

Figure S1. HR-ESI-MS analysis of **1**
Figure S2. NMR analysis of **1**: (A) HMBC, (B) NOESY
Figure S3. HR-ESI-MS analysis of **3** and **4**
Figure S4. HR-MS analysis of **5** and **6**
Figure S5. HR-MS analysis of **2** and its hydrolysis product **7**
Figure S6. NMR analysis of **2** + **7**: (A) HSQC, (B) HMBC, (C) NOESY

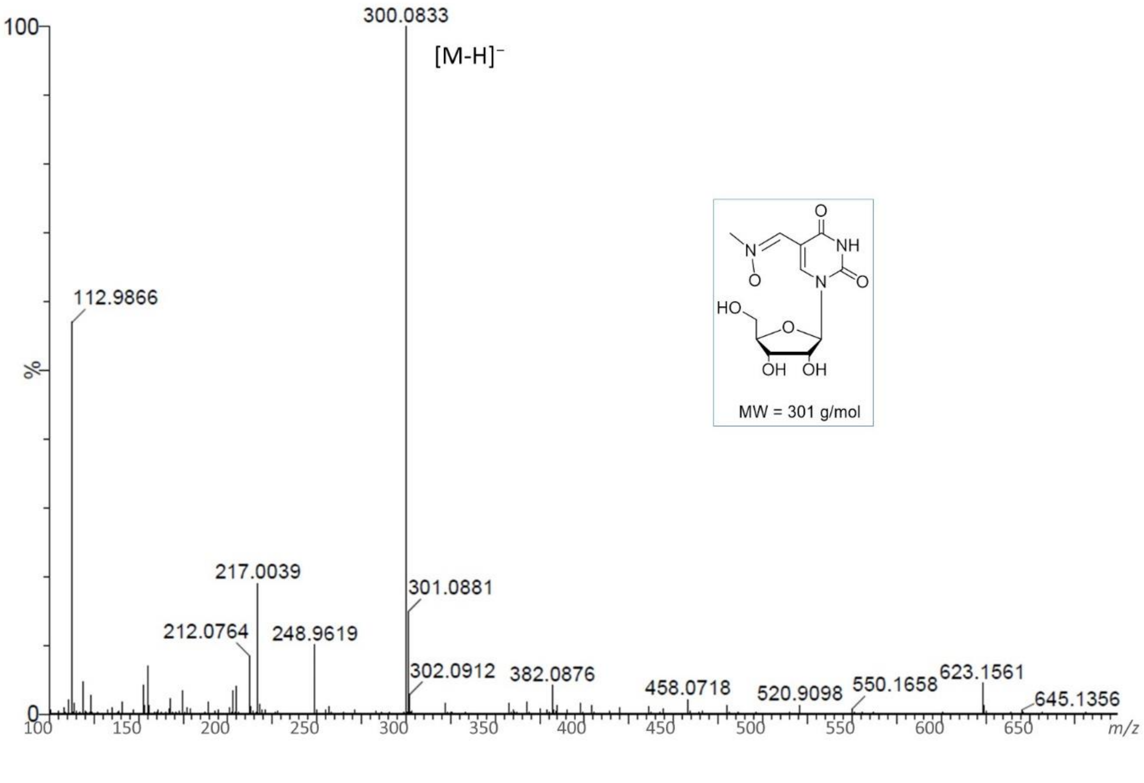

Figure S1. High resolution negative electrospray ionization mass spectrum of the isolated **mnm^{5}U** oxidation product **1**. [M-H]$^-$ signal observed at *m/z* = 300.0833 amu.

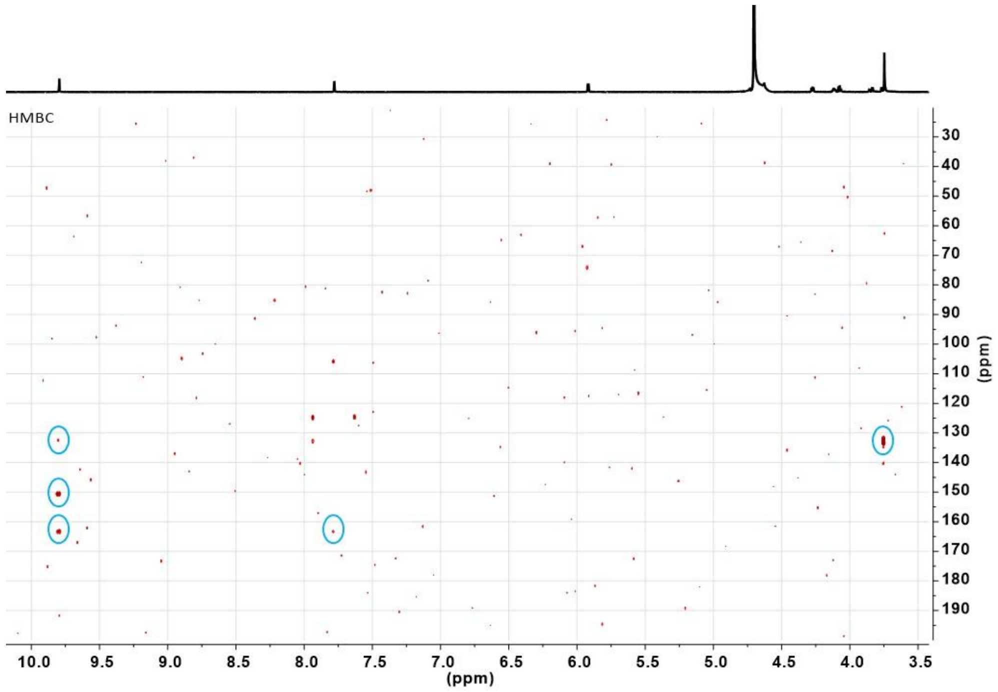

Figure S2_A. HMBC of **1**. Correlation between H signals δ 7.78 and 9.79 ppm with carbon atoms of the nucleobase and correlation between the methyl protons δ 3.75 ppm and the carbon atom of aldonitrone δ 132.5 ppm. 278 K

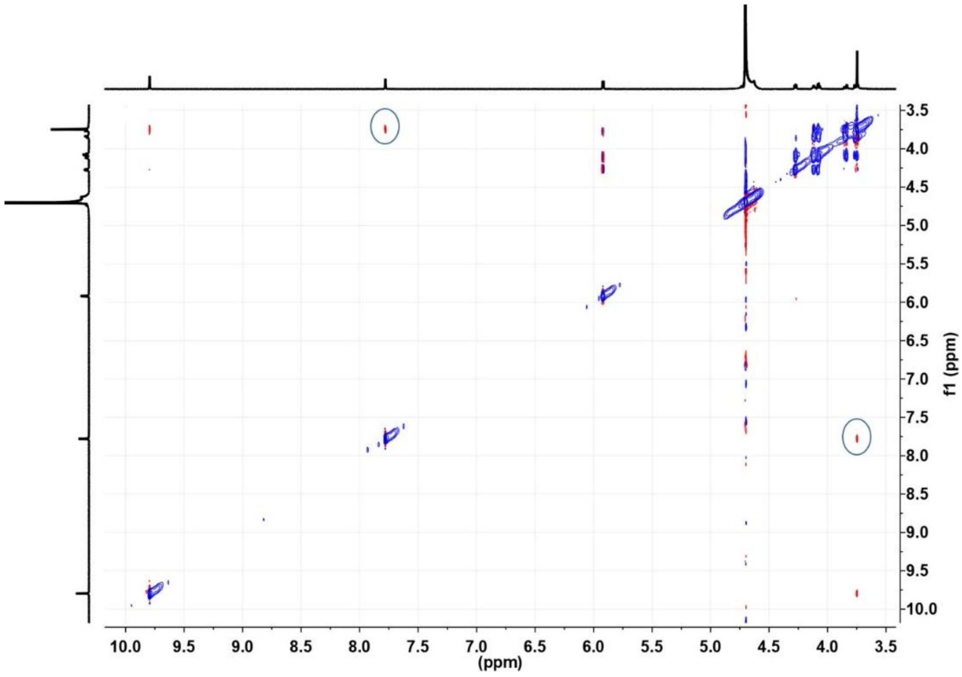

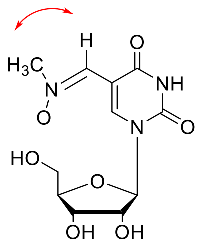

1

Figure S2_B. NOESY between the methyl group δ = 3.75 ppm and the aldonitrone H δ = 7.78 ppm of product **1** in D_2O (blue circle). A correlation between the nucleobase H6 δ = 9.79 ppm and the H5' proton of ribose δ = 3.63 ppm is also observed.

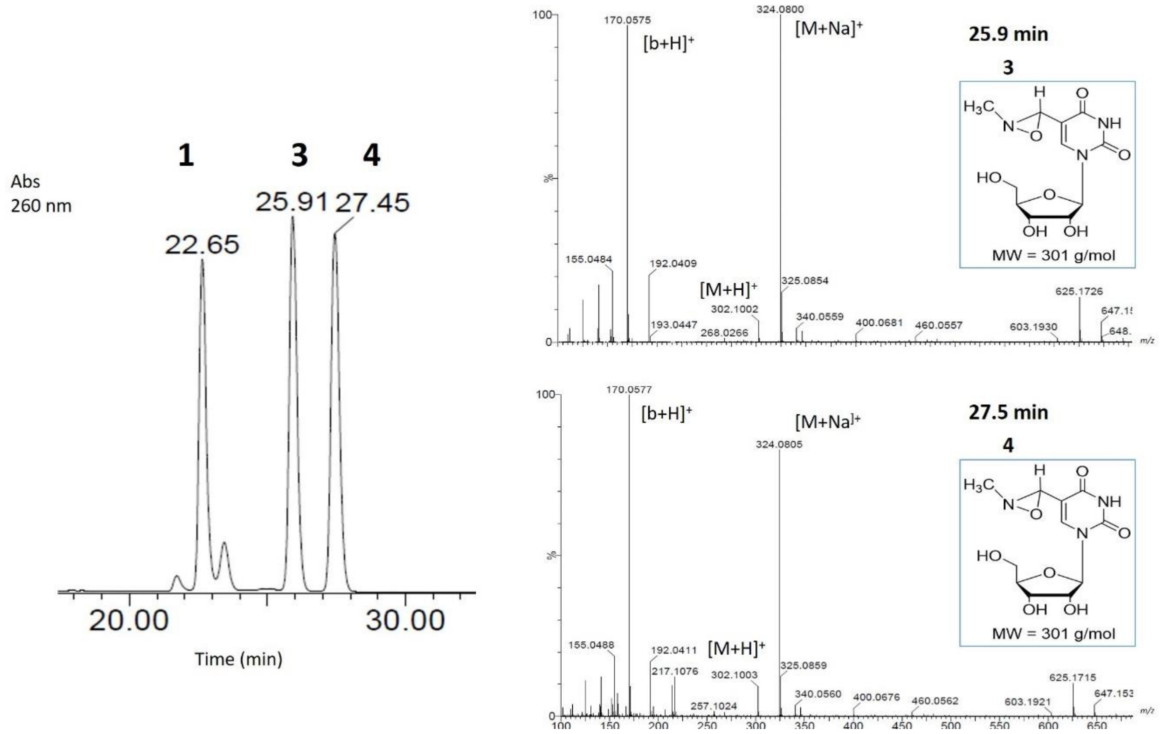

Figure S3. LC/ESI-HR-MS (positive mode) analysis of the irradiation of aldonitrone **1** (Rt = 21.6 min) leading to oxaziridine **3** + **4** mixture. Left: chromatogram, right: in-line mass spectra of **3** and **4**. Sodium adduct of molecular peak $[M+Na]^{+}$ and deglycosylated fragment $[b+H]^{+}$.

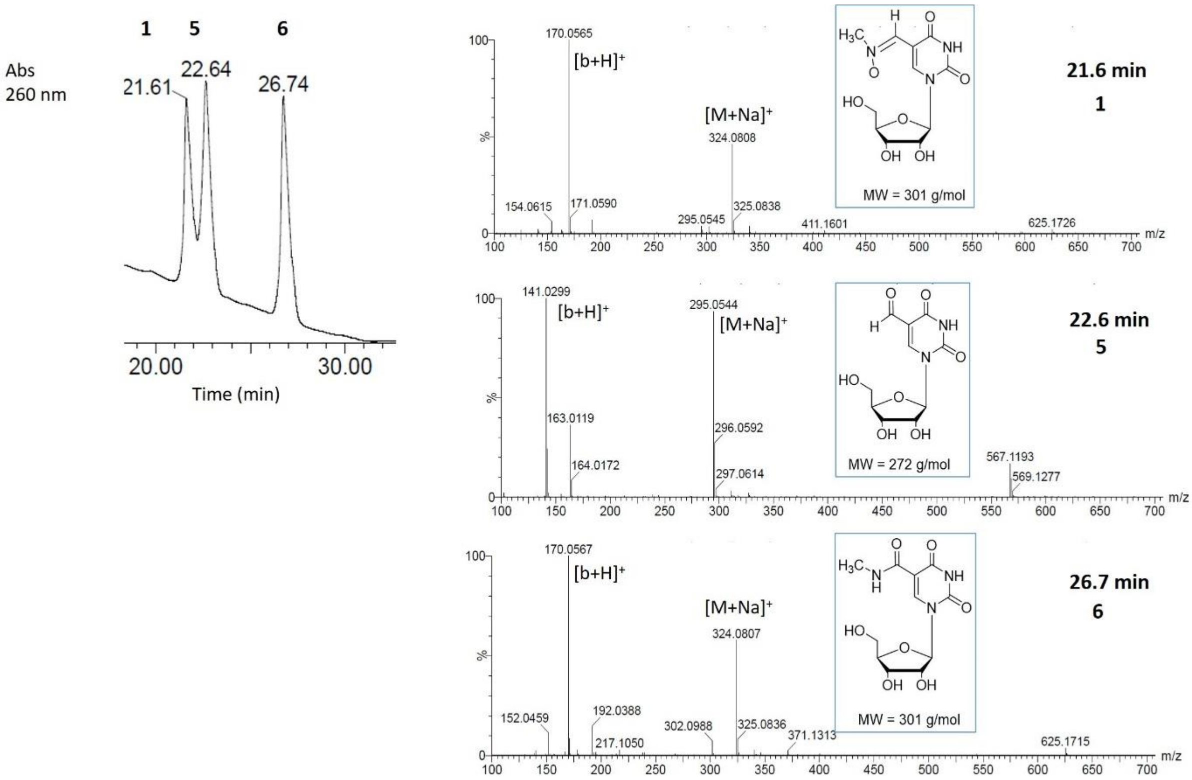

Figure S4. LC/ESI-HR-MS (positive mode) analysis of the reversion of the oxaziridine **3** + **4** mixture after 1 h at 60 °C to aldonitrone **1** (Rt = 21.6 min) with concomitant formation of aldehyde **5** (Rt = 22.6 min), and amide **6** (Rt = 26.7 min) derivatives. Left: chromatogram, right: in-line mass spectra. Sodium adduct of molecular peak $[M+Na]^+$ and deglycosylated fragment $[b+H]^+$.

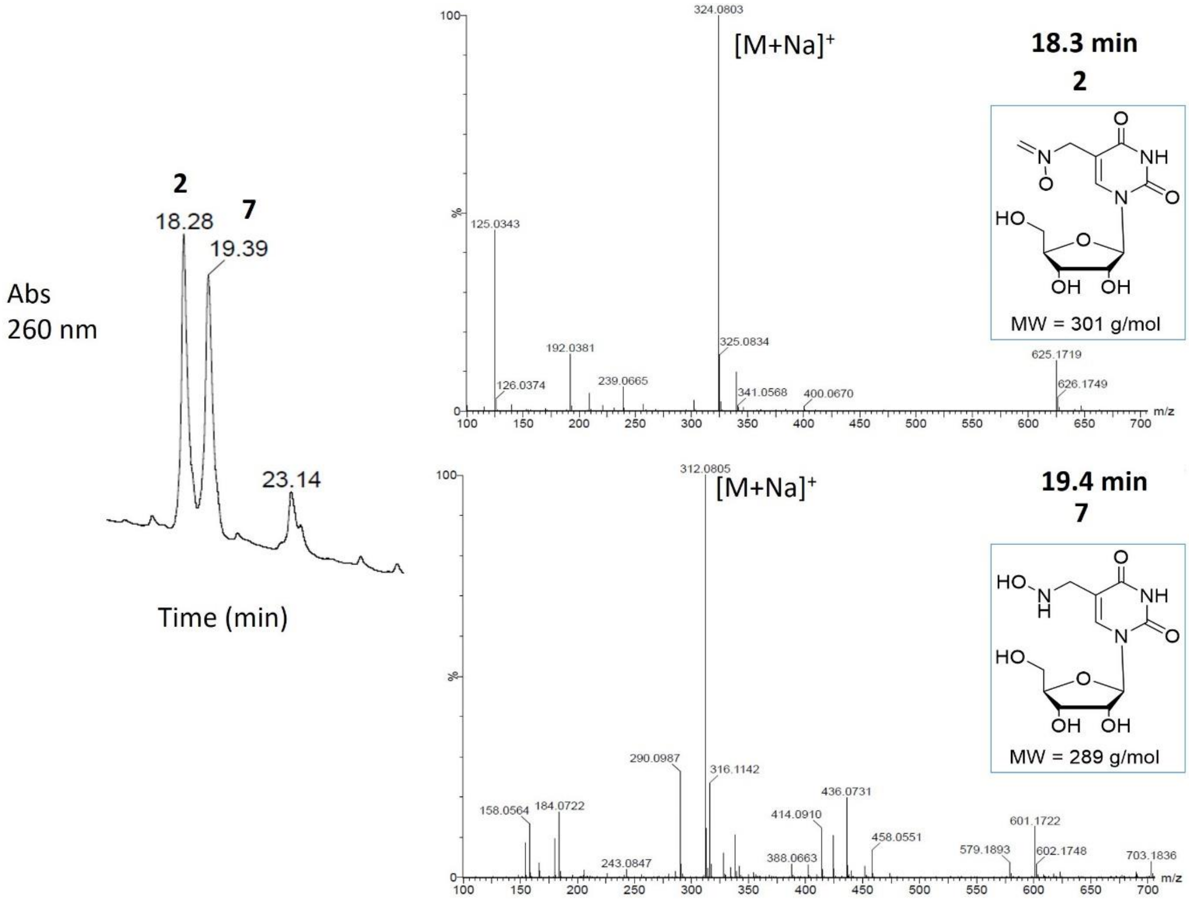

Figure S5. LC/ESI-HR-MS (positive mode) analysis of isolated **2** showing **2** at Rt = 18.3 min and its hydrolysis product, hydroxylamine derivative (**7**) (Rt = 19.4 min). Left: chromatogram, right: in-line mass spectra.

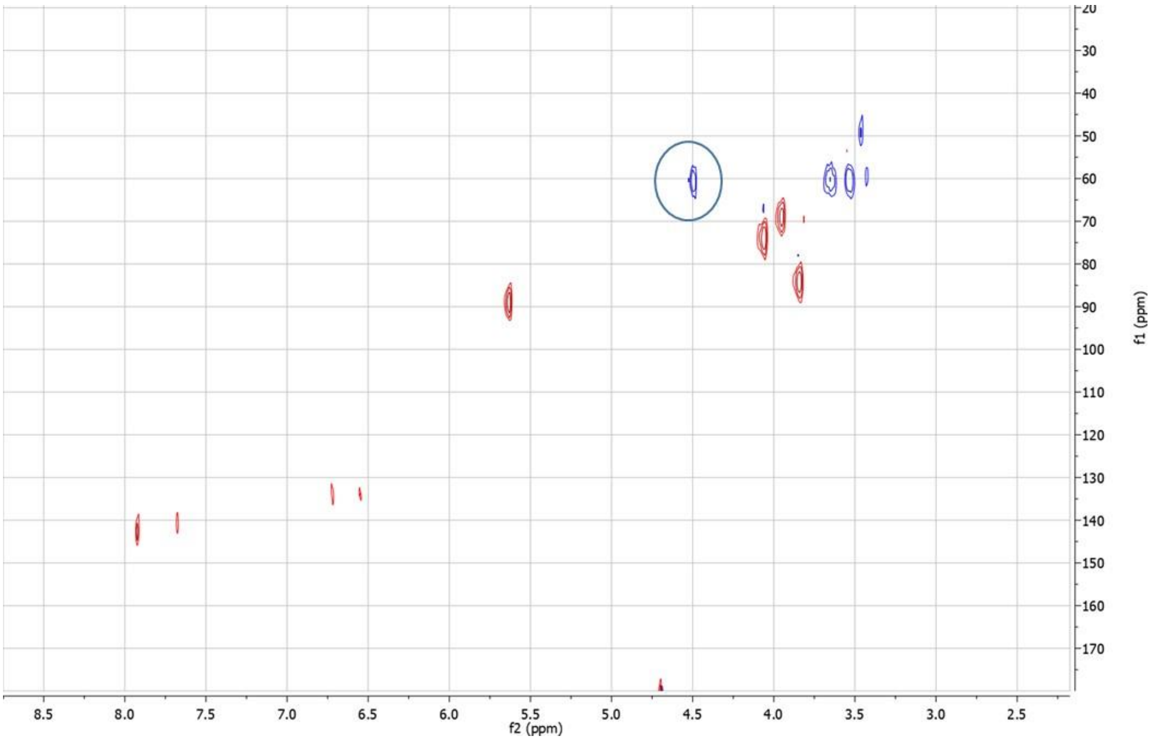

Figure S6_A. Edited-HSQC of the mixture **2** + **7** (^{1}H-NMR spectrum, Figure 13). Correlation between the protons of the CH_2 group at C5 (under the signal of water) and carbon δ 60.5 ppm of product **2** in D_2O is highlighted. Red color refers to CH, CH_3 and blue color to CH_2 protons.

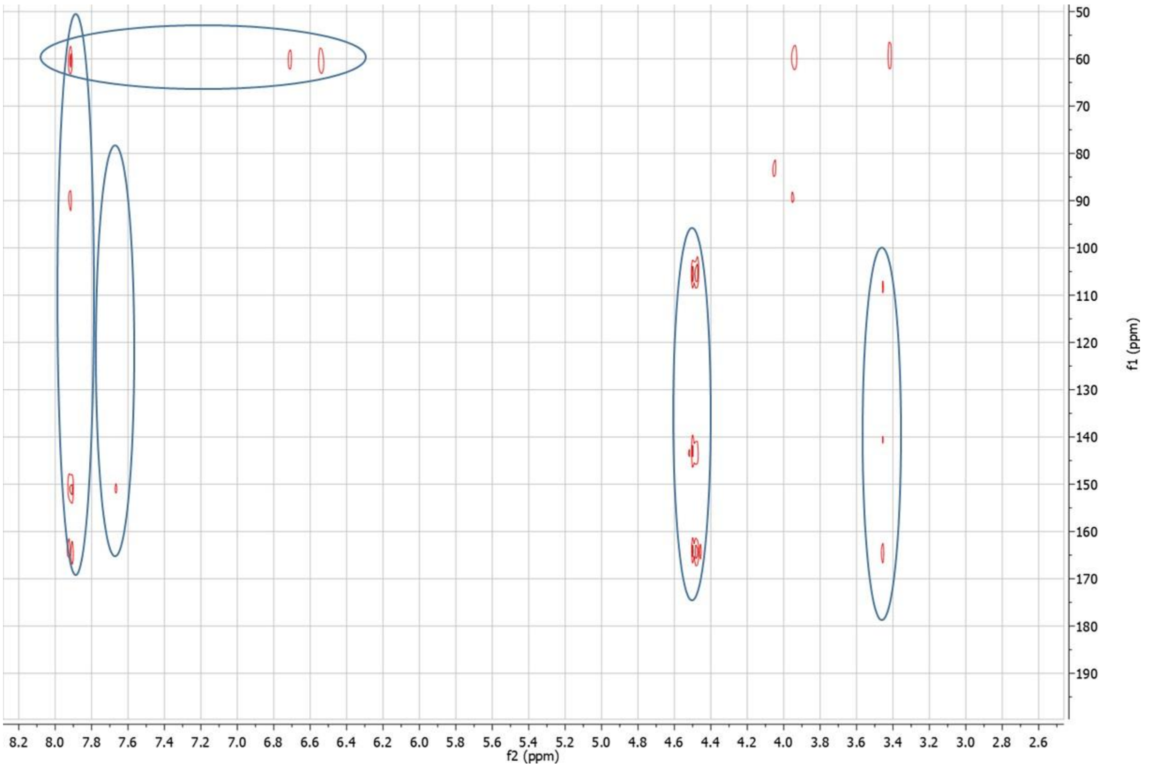

Figure S6_B. HMBC of the mixture of **2** + **7** (corresponding ^{1}H-NMR spectrum: Figure 13).

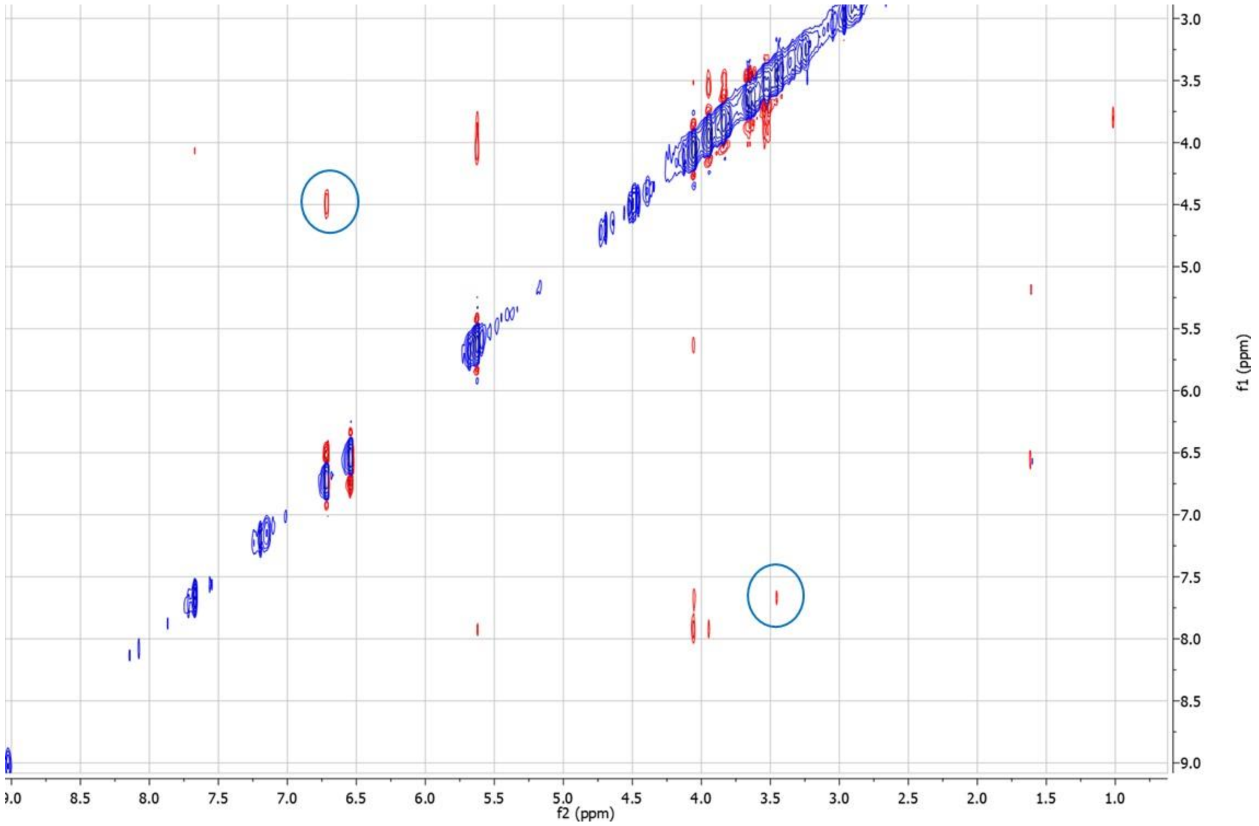

Figure S6_C. NOESY between one of the protons of the terminal methylene of **2** δ 6.70 ppm and the signal under the peak of solvent (δ 4.5 ppm) attributed to the other CH_2 group. Another correlation between the CH_2 protons of **7** δ 3.44 ppm and the nucleobase H6 of **7** δ 7.66 ppm. (corresponding ^{1}H-NMR spectrum: Figure 13).
